# A Multicentric Cross-Sectional Study Investigating the Profile and Motivations of Women Seeking Unsafe Abortions in Yaoundé, Cameroon

**DOI:** 10.7759/cureus.74732

**Published:** 2024-11-29

**Authors:** Michael Bright Fomba Tchoffo, Joel Gabin Konlack Mekontso, Joseph Yvan Bena Nnang, Fabrice Leo Tamhouo Nwabo, Fabrice Ndzernyuy Dubila, Guy Loic Nguefang Tchoukeu, Ulrich Igor Mbessoh Kengne, Yves Alain Notue, Zacharie Sando, Jeanne Fouedjio

**Affiliations:** 1 General Medicine, Higher Institute of Health Sciences, Université des Montagnes, Bangangté, CMR; 2 General Medicine, Faculty of Medicine and Biomedical Sciences, University of Yaoundé I, Yaoundé, CMR; 3 General Medicine, Djeleng Subdivisional Hospital, Bafoussam, CMR; 4 General Medicine, Faculty of Health Sciences, University of Buea, Buea, CMR; 5 General Medicine, Bangou Subdivisional Hospital, Bangou, CMR; 6 General Medicine, Mengueme Subdivisional Hospital, Mengueme, CMR; 7 General Medicine, Faculty of Medicine and Biomedical Sciences, University of Yaounde I, Yaounde, CMR; 8 General Medicine, Messamena Health District Service, Messamena, CMR; 9 Surgery, Eseka District Hospital, Eseka, CMR; 10 Pathology, Faculty of Medicine and Biomedical Sciences, University of Yaoundé I, Yaoundé, CMR; 11 Pathology, Gynaeco-Obstetric and Pediatric Hospital, Yaoundé, CMR; 12 Obstetrics and Gynaecology, Faculty of Medicine and Biomedical Sciences, University of Yaoundé I, Yaoundé, CMR; 13 Obstetrics and Gynaecology, Gynaeco-Obstetric and Pediatric Hospital, Yaoundé, CMR

**Keywords:** cameroon, family planning, female sexual and reproductive health, unsafe abortions, women’s health

## Abstract

Background: Unsafe abortions represent a significant public health issue in Cameroon, often resulting in severe health consequences. This study aimed to investigate the prevalence, motivations, and factors associated with unsafe abortions among women in Yaoundé, Cameroon.

Methods: A cross-sectional study was conducted among women of childbearing age attending three urban health facilities in Yaoundé, Cameroon. Participants provided information on their reproductive history, including any previous abortions. Data on reasons for abortion, methods used, and sociodemographic factors were collected. Regression analysis was performed to identify factors associated with unsafe abortions.

Results: Among 234 women aged 17 to 43, 51 (21.8%) reported at least one unsafe abortion. The primary reasons cited were fear of parental disapproval (24%) and partner refusal (20%). Physicians were the main providers (39.5%), using suction curettage and/or intravaginal misoprostol (89%). Being single (adjusted odds ratio (aOR): 13.6 (5.39-34.4)), nulliparous (aOR: 5.81 (2.41-13.9)), and secondary school students (aOR: 3.23 (1.06-9.81)) were significantly associated with an increased risk of unsafe abortions.

Conclusion: Our study highlights the significant public health problem posed by unsafe abortions in Yaoundé, Cameroon. Single, nulliparous women and secondary school students are at heightened risk. These findings emphasize the urgent need for comprehensive sexual and reproductive health education, increased access to contraception, and safe abortion services targeting these groups.

## Introduction

Abortion is a procedure that terminates a pregnancy before the fetus becomes viable, typically around 28 weeks of gestation in Cameroon [1]. The World Health Organization (WHO) defines an unsafe abortion as one performed by a person lacking the necessary skills or in an environment not meeting minimal medical standards, or both [2]. It is a significant global health issue, contributing to approximately 15% of maternal deaths worldwide [3].

Unsafe abortions disproportionately affect African countries, primarily due to restrictive abortion laws, limited access to reproductive health services, and a high unmet need for family planning [4]. In Cameroon, abortion remains illegal except in cases of rape or incest, forcing many women with unwanted pregnancies to resort to unsafe methods [5,6].

Beyond immediate risks such as hemorrhage and infection, unsafe abortions can result in long-term health consequences, including infertility and various social and psychological issues such as regret, guilt, smoking, alcoholism, and even suicide [7]. To better understand this problem, this study aimed to determine the frequency, reasons, and factors associated with unsafe abortions in Yaoundé, Cameroon.

## Materials and methods

Study design and setting

We conducted a cross-sectional study from January to March 2020 at three public hospitals in Yaoundé, Cameroon: Yaoundé Central Hospital, Biyem-Assi District Hospital, and Efoulan District Hospital. These facilities are high-volume hospitals with specialized obstetric and gynecological services, making them ideal settings for our study.

Study population

Our sample size was estimated at 298 participants using Cochran's formula [8]:

$N = \frac{Z^2 \times p \times (1 - p)}{e^2}$

where N is the sample size, e = 5% is the precision level, Z = 1.96 is a constant, which depends on e, and p = 26.3% is the prevalence of voluntary induced abortion previously reported by Ngowa et al. in Cameroon [9]. Sampling was consecutive, and all consenting women of childbearing age (15-49 years) who came for consultation or hospitalization during the study period were included. Women who had never been pregnant and those with incomplete or missing data were excluded from the study. 

Data collection

Data were collected through confidential, one-on-one interviews. To avoid stigmatizing participants, we used the term "voluntary pregnancy interruption" instead of "abortion." For each reported instance of voluntary pregnancy interruption, we gathered information on the participant's age, marital status, parity, education level, occupation, and contraceptive use at the time of the procedure.

Statistical analysis

Data were analyzed using IBM SPSS Statistics for Windows, Version 23 (Released 2015; IBM Corp., Armonk, New York). The chi-square test and Fisher exact test were used to assess univariate associations. Multivariate logistic regression was performed to identify independent correlates of unsafe abortions. Odds ratios with 95% confidence intervals (OR, 95% CI) were used as measures of association.

Ethical considerations

This study was approved by the Institutional Review Board of the Higher Institute of Health Sciences, Université des Montagnes, Bangangté, Cameroon. Written informed consent was obtained from all participants, and authorization was secured from the managers of the three participating health facilities.

## Results

Prevalence of unsafe abortions, sociodemographic, and reproductive characteristics of respondents

Of the 255 women initially approached, 234 were included in the study after excluding 11 due to missing or incomplete data and 10 who had never been pregnant. Among the 234 women aged 17 to 43, 51 (21.8%) reported having undergone at least one unsafe abortion. All participants identified as Christian, and 45 (88.2%) of those who had undergone an abortion reported that their first sexual experience occurred before the age of 20. Additionally, 21 (41.2%) of these women reported having had more than one abortion, with a total of 81 abortions recorded across the group. We retrospectively gathered information on each participant's social, demographic, professional, and reproductive background. Table 1 highlights the relationship between unsafe abortions and these factors, showing that young, single, childless women under the age of 20 who were students were more likely to have had an abortion. By contrast, married women over the age of 30 with children and stable employment had a lower risk. Further analysis revealed that being single, childless, and in high school were factors significantly associated with higher abortion rates.

**Table 1:** Sociodemographic, reproductive, and professional characteristics of the women surveyed and groups at risk of clandestine abortions. Values in bold represent odds ratios of all independent variables significantly associated with unsafe abortions. OR: odds ratio, CI: confidence interval, aOR: adjusted odds ratio.

Variable	Clandestine abortion		OR (95% CI)	aOR (95% CI)	
	Yes, n (%)	No, n (%)			
Age					
˂20 years	25 (31%)	14 (8%)	5.34 (2.62-11.1)		0.62 (0.18-2.10)
20-30 years	50 (62%)	92 (50%)	1.60 (0.94-2.72)		-
˃30 years	6 (7%)	77 (42%)	0.11 (0.05-0.27)		-
Marital status					
Single	75 (92%)	64 (35%)	23.2 (9.59-56.3)		13.6 (5.39-34.4)
Married	3 (4%)	93 (51%)	0.04 (0.01-0.12)		-
De facto	3 (4%)	25 (13.5%)	0.24 (0.07-0.83)		-
Divorced	0 (0%)	1 (0.5%)	-		-
Parity					
Nulliparous	42 (52%)	12 (6.5%)	15.3 (7.39-31.8)		5.81 (2.41-13.9)
Primiparous	32 (39.5%)	55 (30%)	1.52 (0.88-2.62)		-
Multiparous	7 (8.5%)	116 (63.5%)	0.06 (0.02-0.13)		-
Level of education					
None	0 (0%)	3 (1.5%)	-		-
Primary school	2 (2.5%)	22 (12%)	0.18 (0.04-0.81)		-
Secondary school	52 (64%)	106 (58%)	1.30 (0.76-2.24)		-
University	27 (33.5%)	52 (28.5%)	1.26 (0.72-2.21)		-
Profession					
Secondary schooler	32 (39.5%)	14 (8%)	7.88 (3.89-15.9)		3.23 (1.06-9.81)
University student	19 (23.5%)	19 (10%)	2.65 (1.31-5.33)		-
Employed	14 (17.3%)	85 (46%)	0.24 (0.13-0.46)		-
Housewife	1 (1.2%)	38 (21%)	0.05 (0.01-0.35)		-
Unemployed	15 (18.5%)	27 (15%)	1.31 (0.66-2.63)		-
Contraception					
Yes	9 (11%)	17 (9%)	1.22 (0.52-2.87)		-
No	72 (89%)	166 (91%)	0.82 (0.35-1.92)		-

Motivations of women resorting to unsafe abortions

**Figure 1:**
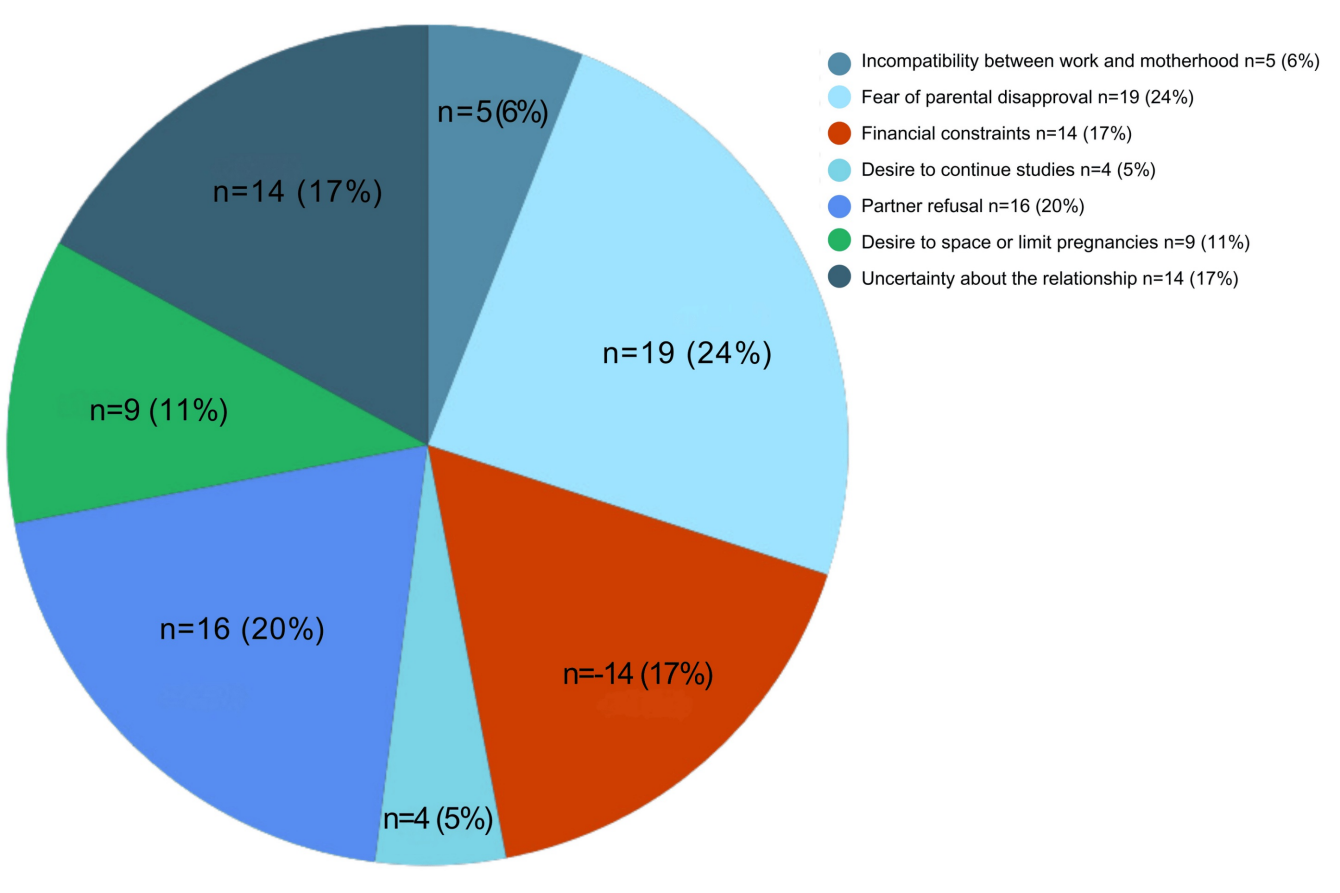
Reasons why women resorted to unsafe abortions.

Figure 1 highlights the reasons cited for seeking unsafe abortions, with the most common being fear of parental disapproval (24%) and partner refusal (20%). Other reasons included financial constraints (17%), uncertainty about the relationship (17%), and unmet family planning needs (11%).

Setting, providers, techniques, and complications of unsafe abortions

Of the 81 reported abortions, 18 (22%) were self-induced at home, while the majority (78%) were performed in healthcare settings, primarily in lower-level facilities. Physicians were the most common providers (32 cases, 39.5%), followed by midwives or nurses (11 cases, 13.5%) and partners (8 cases, 10%). In 30 cases (37%), the women chose not to disclose the identity of the provider. Suction curettage and/or intravaginal misoprostol were the primary methods used (89%). Post-abortion complications included bleeding (16 cases, 20%) and infection (12 cases, 15%). None of the abortions resulted in serious complications. Only 45% (37 cases) of the women received adequate counseling, and just 11 out of the 51 women surveyed (21.5%) left with a reliable contraceptive method.

## Discussion

This study aimed to assess the frequency, underlying reasons, and factors associated with unsafe abortions in Yaoundé, Cameroon. Our findings indicate that unsafe abortions are prevalent among women of reproductive age, with various contributing factors, and that physicians are the most frequent providers. The study also identified a high-risk group that could be prioritized for preventive interventions.

One in five women surveyed reported resorting to abortion, with nearly half having undergone the procedure more than once. Despite legal restrictions and sanctions in Cameroon, the prevalence of unsafe abortions remains high. Other studies in Cameroon have reported similar rates, ranging from 21% to 26.5% [9,10]. This widespread prevalence can be attributed to the low use of modern contraceptives and to social, cultural, and religious barriers that limit access to contraception. Globally, an estimated 40% of pregnancies are unplanned, often due to lack of or improper use of contraception [11,12].

The predominance of Christian participants is likely due to the demographic distribution in Cameroon’s Centre Region, which is mainly Christian, unlike the predominantly Muslim northern regions. Additionally, most respondents were students, as Yaoundé, with its numerous high schools and universities, attracts young people from other parts of the country. Although age under 20 years was significantly associated with abortion in univariate analysis, this association did not hold in multivariate analysis, possibly due to our limited sample size. Overall, unsafe abortions were more common among single, childless secondary school students. This finding may be attributed to several factors: a high proportion of young women are sexually active by their late teens (with over 88% of those surveyed being sexually active before the age of 20), often in short-term relationships, and remain financially dependent on their parents. Many want to continue their studies and avoid the stigma of out-of-wedlock pregnancy due to parental and societal disapproval. These circumstances align with the reasons they cited for seeking abortions. Moreover, Cameroon's secondary school curricula include minimal or no sexual education, which likely contributes to unplanned pregnancies and, subsequently, abortions.

The age range of women seeking abortions varies widely in the literature. A study from Ghana found particularly high abortion rates among women under the age of 20 [13], whereas other studies report that nearly half of all abortions worldwide occur among women aged 20-30 years [14]. Similarly, Adjei et al.'s study in Ghana identified single status as significantly associated with induced abortions [13], while Calvès et al. reported a high rate of abortion among single women in Cameroon, reaching 76.3% [15]. Further studies conducted in Ghana in 2014 and 2017 found that nulliparity [14] and student status [16] were also significantly linked to higher abortion rates.

Abortions were performed either at home or in small health facilities by medical personnel. Due to restrictive abortion laws, women often turn to providers with limited skills or to facilities lacking basic medical standards. Additionally, challenging socioeconomic conditions have led some health workers to view abortion as an opportunity to earn extra revenue.

Suction curettage and misoprostol were the primary methods used for abortion (89%), likely because physicians were the main providers and most were trained in these techniques. Similar findings were reported by Ngowa et al. in Cameroon [9]. Additionally, Wonkam and Hurst noted an increasing acceptance of voluntary abortion among medical trainees in Cameroon, with acceptance rising from preclinical students to clinical students and then to practicing physicians [17].

Out of the 51 women surveyed, 18 (35%) reported complications, primarily bleeding and infection, findings consistent with those of other studies [9,10]. Most complications occurred in women who had undergone abortion at home. Fortunately, all cases resolved without any serious outcomes.

In Cameroon, strong social, cultural, and religious barriers to contraception lead many adolescents and young women to rely on abortion as an alternative. This issue is further exacerbated by the neglect of some healthcare providers in offering adequate post-abortion care. Less than half of the women who underwent abortion received proper counseling, and only one in five left with a reliable contraceptive method. By contrast, Madziyire et al. reported that 92% of women in their Zimbabwean study received family planning counseling, and 43% were discharged with a modern contraceptive method, possibly because most of their patients (80%) were married. Married women may demonstrate greater maturity, responsibility, and adherence to family planning methods [18].

Our study is subject to several limitations. The women surveyed may have concealed their past resort to abortion because of the sensitive nature of the topic and the illegality of the procedure in Cameroon. This may have underestimated the true prevalence of unsafe abortions. Additionally, we were unable to assess long-term complications such as infertility, which would require a longitudinal study design.

## Conclusions

Unsafe abortion remains a significant public health concern in Cameroon. To effectively address this issue, a comprehensive approach is needed, involving collaboration among families, policymakers, and health and education stakeholders. Prioritizing life skills, relationship education, and sexual health programs, particularly for young girls, is essential to reducing the prevalence of unsafe abortions.
